# Tumor-selective use of DNA base excision repair inhibition in pancreatic cancer using the NQO1 bioactivatable drug, β-lapachone

**DOI:** 10.1038/srep17066

**Published:** 2015-11-25

**Authors:** Gaurab Chakrabarti, Molly A. Silvers, Mariya Ilcheva, Yuliang Liu, Zachary R. Moore, Xiuquan Luo, Jinming Gao, Glenda Anderson, Lili Liu, Venetia Sarode, David E. Gerber, Sandeep Burma, Ralph J. DeBerardinis, Stanton L. Gerson, David A. Boothman

**Affiliations:** 1 Departments of Pharmacology, Dallas, TX 75390-8807; 2 Radiation Oncology, Simmons Comprehensive Cancer Center, UT Southwestern Medical Center, Dallas, TX 75390-8807; 3 5 Degrees Bio, San Jose, CA 95113; 4 Department of Hematology and Oncology, Case Western Reserve Comprehensive Cancer Center, Cleveland, OH 44106; 5 Department of Pathology, UT Southwestern Medical Center, Dallas, TX 75390-9234; 6 Children’s Medical Center Research Institute, UT Southwestern Medical Center, Dallas, TX75390-8502.

## Abstract

Base excision repair (BER) is an essential pathway for pancreatic ductal adenocarcinoma (PDA) survival. Attempts to target this repair pathway have failed due to lack of tumor-selectivity and very limited efficacy. The NAD(P)H:Quinone Oxidoreductase 1 (NQO1) bioactivatable drug, ß-lapachone (ARQ761 in clinical form), can provide tumor-selective and enhanced synergy with BER inhibition. ß-Lapachone undergoes NQO1-dependent futile redox cycling, generating massive intracellular hydrogen peroxide levels and oxidative DNA lesions that stimulate poly(ADP-ribose) polymerase 1 (PARP1) hyperactivation. Rapid NAD^+^/ATP depletion and programmed necrosis results. To identify BER modulators essential for repair of ß-lapachone-induced DNA base damage, a focused synthetic lethal RNAi screen demonstrated that silencing the BER scaffolding protein, XRCC1, sensitized PDA cells. In contrast, depleting OGG1 N-glycosylase spared cells from ß-lap-induced lethality and blunted PARP1 hyperactivation. Combining ß-lapachone with XRCC1 knockdown or methoxyamine (MeOX), an apyrimidinic/apurinic (AP)-modifying agent, led to NQO1-dependent synergistic killing in PDA, NSCLC, breast and head and neck cancers. OGG1 knockdown, dicoumarol-treatment or NQO1- cancer cells were spared. MeOX + ß-lapachone exposure resulted in elevated DNA double-strand breaks, PARP1 hyperactivation and TUNEL+ programmed necrosis. Combination treatment caused dramatic antitumor activity, enhanced PARP1-hyperactivation in tumor tissue, and improved survival of mice bearing MiaPaca2-derived xenografts, with 33% apparent cures. Significance: Targeting base excision repair (BER) alone has limited therapeutic potential for pancreatic or other cancers due to a general lack of tumor-selectivity. Here, we present a treatment strategy that makes BER inhibition tumor-selective and NQO1-dependent for therapy of most solid neoplasms, particularly for pancreatic cancer.

Pancreatic ductal adenocarcinoma (PDA) is predicted to be the second leading cause of cancer-related death in the US by 2020, with a current 5-year survival rate of <6%^1^. The majority of chemotherapies approved for the therapy of PDA include DNA-damaging agents (ionizing radiation, cisplatin, irinotecan) or antimetabolites that inhibit DNA synthesis (5-fluorouracil, gemcitabine)^2^. These agents have very narrow therapeutic windows, with tumor selectivity based solely on differential tumor replication rates, and are consequently highly toxic to normal tissues with high turnover rates in patients. There is a desperate need to identify new therapeutic targets and strategies to specifically target PDAs, while sparing normal tissue.

Novel NAD(P)H:quinone oxidoreductase 1 (NQO1, EC1.6.99.2) bioactivatable drugs represent a promising new tool for the novel tumor-selective treatment of PDAs. NQO1 is elevated at levels ranging from 5- to 100-fold in PDAs above associated normal pancreas tissue^3^. The two-electron oxidoreductase is an inducible phase II detoxifying enzyme capable of detoxifying quinones by forming stable hydroquinones. These are, in turn, conjugated with glutathione via the action of glutathione S transferase and excreted from the cell^4^. For most quinones, this reaction avoids toxic one-electron oxidoreductions in normal cells. However, in recent years our laboratory discovered specific novel quinones that undergo futile redox cycling by NQO1^5^. The NQO1 bioactivatable drug, ß-lapachone (ß-lap, in clinical form, ARQ761), is metabolized by NQO1 to form an unstable hydroquinone that spontaneously oxidizes back to the parent compound in two one-electron oxidations, consuming two separate oxygen molecules. This reaction generates a futile redox cycle whereby one mole of ß-lap generates ~120 moles of superoxide within two minutes consuming ~60 moles of NAD(P)H^6^. Superoxide (O_2_^.-^) radicals are quickly metabolized by superoxide dismutase (SOD) into hydrogen peroxide (H_2_O_2_)^7^. Elevated, cell membrane-permeable H_2_O_2_ pools, in turn, lead to extensive oxidative DNA lesions, particularly base and DNA single strand breaks, that ‘hyperactivates’ poly(ADP-ribosyl) polymerase 1 (PARP1). The net result is that the elevated levels of NAD^+^ created by NQO1 futile redox cycling of ß-lap are subsequently degraded by hyperactivated levels of PARP1. As a result, rapid and dramatic depletion of intracellular NAD^+^ pools occur within 20–30 mins of ß-lap exposure in NQO1+ cancer cells^8^. Consequentially, ATP levels rapidly and dramatically fall explaining the earlier action of this drug to inhibit the repair of ß-lap-induced or DNA damage (e.g., ionizing radiation)-induced DNA lesions^9^, as well as all other downstream effects reported for this drug^6^. ß-Lap-exposed, NQO1+ cancer cells die by a unique caspase-independent programmed necrosis pathway, termed NAD^+^-Keresis^8^. Few drugs mechanistically act to induce PARP1-mediated programmed necrosis in a tumor-specific manner at clinically-relevant doses^10^. Most agents known to stimulate PARP1-mediated programmed necrosis (e.g., >5 mM MNNG or >0.3 mM hydrogen peroxide (H_2_O_2_))^11^,^12^ do so at supra-lethal, clinically non-achievable doses. Cancer cells with >100 units of NQO1 enzyme activity are hypersensitive to ß-lap, while normal tissues that lack, or express low levels of, NQO1 are spared^13^.

We previously demonstrated that a lethal dose of ß-lap (~4 μM) creates a significant level of DNA lesions in NQO1-expressing cancer cells immediately after drug exposure^9^. However, when cells were analyzed by neutral comet assays, DNA double strand breaks (DSBs) were not initially detected. In contrast, alkaline comet assays revealed that the majority of DNA lesions immediately created in ß-lap-exposed NQO1+ cancers were DNA base and single strand break (SSB) lesions^9^. Since PARP1 can bind to DNA base apyrimidinic/apurinic (AP) sites and SSBs^14^,^15^,^16^, upstream base excision repair (BER) processes represent possible ‘targetable’ resistance mechanisms that when inhibited might improve the efficacy of NQO1 bioactivatable drugs, such as ß-lap. Such a strategy may lower dose-limiting methemoglobinemia (MH) caused by NQO1 bioactivatable drugs, while enabling, for the first time, tumor-selective use of DNA repair, specifically BER, inhibitors. This is especially important since PDAs appear to heavily rely on BER for genomic sanitization in the face of increased mutagenic loads from hypoxia-induced oxidative base damage^17^,^18^.

BER removes and repairs non-helix distorting base lesions in the genome. Unlike nucleotide excision repair (NER), which repairs helix-distorting DNA lesions, BER is essential for removing oxidatively damaged bases that can cause genomic mutations through base mispairing or replicative-associated DNA breaks^19^. Bases in DNA can be damaged by a variety of mechanisms, including oxidation, deamination and alkylation. One of the most common base lesions resulting from ROS exposure is the oxidation of the 8th carbon of guanine, forming 8-oxoguanine (8-oxo-G)^20^. 8-Oxo-G formation can result in the mismatched pairing of guanine to adenine, causing G:C to G:A transversions, thereby compromising a cell’s genomic integrity leading to a pro-oncogenic environment. However, cells evolved DNA N-glycosylases, such as OGG1, as dedicated repair enzymes to detect and cleave 8-oxo-G from the DNA backbone. Once 8-oxo-G lesions are excised by OGG1, as well as by other less avid DNA N-glycosylases, AP sites are generated. PARP1 can bind AP sites, which in turn, stimulates its poly(ADP-ribose) polymerase activity stimulating poly-ADP-ribosylation using NAD^+^ as a substrate. AP sites are also substrates for AP endonuclease 1 (APE1) or AP lyase, and these enzymes can generate SSBs in the DNA backbone^14^,^15^,^16^,^19^,^21^,^22^,^23^ that also serve as substrates for PARP1. Through PARylation of downstream proteins, active PARP1 stimulates DNA ligase IV and recruits the BER scaffolding protein, XRCC1, that facilitates localization of BER complexes that resolve DNA damaged sites. Auto-PARylation of PARP1 facilitates dissociation of PARP1 from damaged sites, allowing DNA repair to proceed, while PAR-PARP1 is rejuvenated by poly(ADP-ribose) glycohydrolase (PARG)^15^,^24^,^25^ generating free PAR moieties.

Here, we show that the expression profile of NQO1 and Catalase levels in PDA tumors offers an optimal therapeutic window for use of NQO1 bioactivatable drugs alone, or in combination with a BER inhibitor (e.g., methoxyamine (MeOX)) to afford synergistic antitumor responses. At lethal doses of the drug, the massive pools of H_2_O_2_ generated from NQO1-dependent ß-lap futile redox cycling and the concomitant lowered levels of Catalase in PDA tumors, leads to induction of oxidative DNA lesions, including extensive 8-oxo-G formation. At sublethal doses of ß-lap that cause DNA lesions, but no visible 8-oxo-G, co-administration of MeOX can convert normally repaired AP sites to lesions that hyperactivates PARP1 and ultimately cell death through a form of programmed necrosis, called NAD^+^-Keresis^8^. We conducted a focused BER-specific small interfering RNA (siRNA) synthetic lethal screen that revealed X-ray repair cross-complementing group 1 (XRCC1) and 8-oxo-G DNA N-glycosylase 1 (OGG1) as genes that modulate PARP1 hyperactivation and ß-lap efficacy. Therapeutically, we demonstrated that addition of the AP site-modifying drug and BER small molecule inhibitor, MeOX, to ß-lap greatly potentiated antitumor efficacy and survival of PDA xenograft-bearing mice, with an apparent cure rate of 33%. The drug combination is beneficial to both agents, with ß-lap affording tumor-selectivity to MeOX, and MeOX lowering the efficacious dose of ß-lap required for antitumor activity and survival of PDA-bearing mice. This strategy avoids dose-limiting hemolysis and MH noted with ß-lap (ARQ761 in clinical form). This combination is the first reported regimen that offers tumor-selective use of BER inhibitors.

## Results

### Tumor vs normal tissue NQO1:Catalase expression ratios offer an exploitable therapeutic window for pancreatic cancer

**Figure 1:**
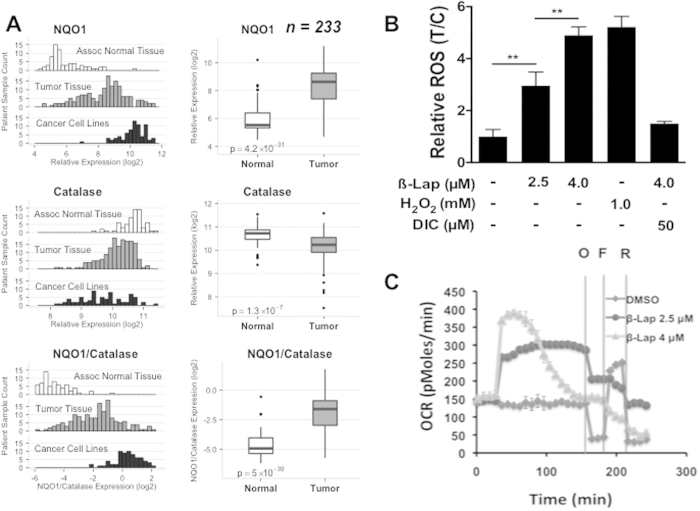
NQO1:Catalase ratios are elevated in PDA tumor vs normal pancreatic tissue. (**A**) NQO1, Catalase and NQO1:Catalase ratio expression was evaluated in 164 PDA tumor, and 69 normal pancreatic tissue samples. NQO1, Catalase and NQO1:Catalase expression levels from 73 PDA cancer cell lines are also shown for comparison; (**B**) ROS levels formed in 30 mins were assessed in MiaPaca2 cells treated with H_2_O_2_, or ß-lap, with or without dicoumarol (50 μM) at the indicated doses using CellRox-Glo. Values were normalized to DMSO-treated control cells. **(C)** Oxygen consumption rates (OCRs) in MiaPaca2 cells treated with 2.5 μM or 4 μM ß-lap ± DIC followed by mitochondrial inhibition with oligomycin (**O**), FCCP (F), and rotenone (**R**). Data suggest that OCR and ROS formation in response to ß-lap are NQO1-dependent.

Analyses of mRNA expression data from 164 pancreatic tumor samples, 69 normal pancreatic ductal tissue (a total of 233 specimen), as well as 73 pancreatic cancer cell line specimens for comparison, revealed that NQO1 mRNA levels were elevated 5- to >100-fold in pancreatic tumor samples compared to normal tissue (Fig. 1A). Interestingly, corresponding Catalase mRNA levels were significantly lower in PDA *vs* associated normal pancreatic tissue, with NQO1:Catalase ratios significantly elevated in PDA (Fig. 1A). In contrast, associated normal tissue expressed relatively elevated Catalase levels with concomitantly low levels of NQO1 (Fig. 1A). Catalase can be an important downstream resistance factor in normal tissue against NQO1 bioactivatable drugs, such as ß-lapachone (ß-lap)^6^. In contrast, in PDA cancers with elevated NQO1 levels (>100 Units), Catalase expression has a lesser effect as the massive H_2_O_2_ levels created after ß-lap exposure could overwhelm typically lowered Catalase levels (Fig. 1A)^13^. Within the database, 59 directly matched tumor *versus* normal tissue samples also showed the same elevated NQO1:Catalase ratio (**SF1**). Thus, NQO1:Catalase ratios in PDA tumor *vs* associated normal pancreas tissue potentially offer a significant therapeutic window that may be exploited using unique NQO1 bioactivatable drugs, such as ß-lap (ARQ761). This may be particularly true for the protection of normal tissue, where lack of NQO1 means extremely low level futile redox cycling, while elevated Catalase levels should offer protection as we previously showed for breast cancer xenografts^26^. We also noted the divergence of human pancreatic cancer cell lines, where NQO1 levels were substantially higher than in tumor tissue and Catalase levels are extremely varied (Fig. 1A). MiaPaca2 and other cancer cells examined were chosen because they matched the average NQO1 and Catalase expressions in tumors noted in Fig. 1A.

### ß-Lap exposure induces NQO1-dependent cytoplasmic ROS stress, resulting in significant DNA base damage

**Figure 2:**
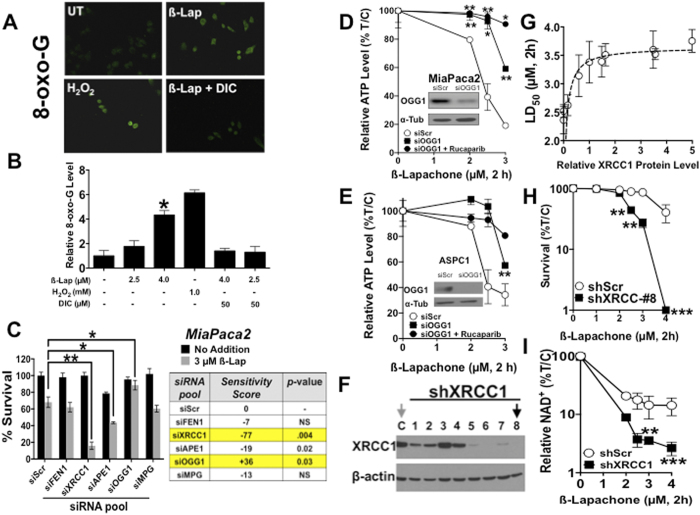
OGG1 and XRCC1 modulate cellular responses to ß-lap. (**A**) Relative 8-oxo-G levels were monitored in MiaPaca2 cells before or after ß-lap (4.0 μM), with or without co-administration of dicoumarol (50 μM) and compared to DMSO-treated control cells after 30 min treatments (DAPI, SF3A). **(B)** Relative 8-oxo-G levels (Intensity Quantification) of MiaPaca2 cells treated as in **A** using NIH ImageJ after exposure to various ß-lap doses, with or without dicoumarol, vehicle alone or H_2_O_2_ at indicated doses. **(C)** MiaPaca2 cells were transfected with pooled RNAis against specific BER proteins and treated 48 h later with DMSO or ß-lap (3 μM, 2 h) and survival was assessed using colony forming assays. XRCC1 and OGG1 were identified as genes altering sensitivity to ß-lap. **(D,E)** OGG1 protein levels were depleted in 48 h by specific siRNAs in MiaPaca2 or ASPC1 cells. Cells were then treated with ß-lap ± Rucaparib (25 μM) for 2 h and ATP levels monitored using CellTiter-Glo assays. (**F**) Stable shXRCC1 knockdown MiaPaca2 clones were generated as assessed by Western immunoblotting. (**G**) Clonogenic survival assays were used to determine LD_50_ values for each ß-lap-treated MiaPaca2-non-targeting (NS) or shXRCC1 clones in separate dose-response studies. Plating efficiencies were not altered by shXRCC1 depletion. (**H**) ß-Lap dose-response of shXRCC1 MiaPaca2 clone #8 by clonogenic survival assays. (**I**) Relative intracellular NAD^+^ levels in stable shScr or shXRCC1 knockdown MiaPaca2 cells before and after exposure to various doses of ß-lap (μM, 2h). Results were compared using Student’s t-tests (+/− standard deviations). *p *<* 0.05; **p *<* 0.01; ***p *<* *0.001.*

Prior data from our laboratory demonstrated that a 30–60 min exposure to ß-lap at 4 μM, but not 2.5 μM, could kill NQO1+-overexpressing cancer cells, including PDA MiaPaca2 cells. Understand DNA lesions created by sublethal doses of ß-lap, such as ROS-induced DNA base damage and SSBs could allow conversion of sublethal damage to lesions, such as DSBs, that cause lethality (**SF2A**). Cells die by a PARP1 hyperactivation programmed necrosis mechanism of action, regardless of p53, anti-apoptotic factor, or cell cycle statuses^8^,^13^,^27^,^28^,^29^,^30^. Understanding ROS stress and DNA damaging events during this ‘minimum time to death’ period is critical for delineating essential mechanisms for improving efficacy of this clinically-relevant (i.e., ARQ761) drug. Total ROS formation during the first 30 min of ß-lap treatment in MiaPaca2 cells was NQO1-specific and dose-dependent, with 4 μM ß-lap (a lethal dose in MiaPaca2 cells) causing ROS levels that were approximately equivalent to a supra-lethal dose of H_2_O_2_ (1 mM, 30 mins) (Fig. 1B); the LD_90_ of exogenously administered H_2_O_2_ treatment of MiaPaca2 cells was ~20 μM under the same conditions, which does not cause PARP1 hyperactivation^13^. Importantly, the NQO1 inhibitor, dicoumarol (Dic), completely suppressed ß-lap-induced ROS formation (Fig. 1B), while Dic did not affect lethality caused by exogenous H_2_O_2_^9^,^13^,^26^. Cells treated with 4.0 μM ß-lap displayed a short-term dramatic burst in O_2_ consumption rate (OCR), while exposure to a sublethal dose of ß-lap (2.5 μM) caused lower but sustained OCRs that did not decrease over time as noted after 4.0 μM ß-lap exposure (Fig. 1C). Additionally, increases in OCRs by ß-lap were independent of mitochondrial function, since suppressing mitochondrial function with oligomycin (O) and rotenone (R) pretreatments (**SF2B**) did not prevent drug-induced OCRs. Over time, 4 μM ß-lap-induced OCR decreased consistent with losses of NAD(P)H, NAD(P)+ and ATP, previously noted at >60 mins post-treatments^13^,^29^. Importantly, elevated ß-lap-induced OCR was blocked by Dic, consistent with an NQO1-dependent mechanism of O_2_ utilization and ROS formation in the cytoplasm where NQO1 is intracellularly located. As a result of massive ß-lap-induced OCR and ROS formation (Fig. 1B), dramatic increases in 8-oxo-G DNA base lesion formation were noted in a dose- and NQO1-dependent manner, as administration of Dic greatly suppressed these DNA lesions (Fig. 2A,B). Along with DNA single strand breaks (SSBs)^7^,^27^, 8-oxo-G moieties are the prominent DNA lesions generated by ß-lap, with minimal DNA double strand breaks (DSBs) generated in a delayed manner^29^. Importantly, while exposure of cells to 2.5 μM ß-lap did not induce a significant steady state level of 8-oxo-G moieties in DNA (Fig. 2B), cells exposed to this sublethal dose induced significant and sustained OCRs (Fig. 1C) that generated significant ROS formation (Fig. 1B) and total DNA damage (DNA base and SSBs) noted by alkaline comet assays (**SF2C**). In contrast, DNA DSBs noted by neutral elution comet assays at the same dose were not initially induced by exposure to 2.5 μM ß-lap (**SF2C**). These ‘total’ alkaline-labile sites and SSBs are key to the PARP1 hyperactivation mechanism of action of this agent, and indicated an essential mechanism by with BER inhibition could be exploited for improved therapy^28^. The lack of 8-oxo-G in DNA after 2.5 μM ß-lap strongly suggest that OGG1 and BER were able to process the damage and prevent steady state level increases, resulting in AP sites and SSBs that are rate-limiting in cells exposed to this dose of drug.

### OGG1 and XRCC1 are key modulators of ß-lap-induced PARP1 hyperactivation and lethality

To identify BER factors essential for repair of ß-lap-induced DNA base and SSB lesions, a pooled RNAi screen against a limited and focused number of BER proteins was performed in NQO1+ MiaPaca2 PDA cells, including: FLAP endonuclease 1 (FEN1), XRCC1, AP endonuclease 1 (APE1), OGG1 and methyl-purine-DNA glycosylase (MPG) genes/proteins. Sensitivity was determined using an ~LD_40_ dose of ß-lap (3 μM, 1 h) in siScr MiaPaca2 cells for each siRNA pool (4 siRNAs/gene) (Fig. 2C). The influence of depletion of each gene was reflected in a sensitivity score, defined as the ratio of %*Survival at 3* *μM with knockdown* to %*Survival with* siScr, subtracted by 1 and multiplied by 100 (thus, siScr = 0, a negative score = sensitizer, and a positive score = non-sensitizer or protector. Importantly, OGG1 depletion rescued cells from ß-lap-induced lethality, with a sensitivity score of +36. In contrast, depletion of XRCC1, an essential scaffolding protein for both long and short patch BER, dramatically sensitized cells to ß-lap, with a sensitivity score of −77 (Fig. 2C). OGG1 was silenced using two different sources of OGG1-specific siRNAs and knockdown was confirmed by Western blot analyses in MiaPaca2 and ASPC1 PDA cells (**insets,** Fig. 2D,E). Minimal effects were noted with siFEN1 knockdown, while siAPE1 depletion resulted in some level of sensitization of MiaPaca2 cells to ß-lap. Collectively, these data are consistent with prolonged AP sites and/or SSBs as the primary DNA lesions that sensitize NQO1+ PDA cells to ß-lap-induced PARP1 hyperactivation-mediated lethality.

Since OGG1 detects and cleaves 8-oxo-G moieties from the DNA backbone to generate APE1- or AP lyase-susceptible AP sites that, in turn, are detected and bound, by PARP1^6^,^14^, we determined whether OGG1 loss reduced ß-lap-induced PARP1 hyperactivation, NAD^+^/ATP loss and lethality. Total intracellular ATP levels were measured 2 h after ß-lap treatment as a proxy for PARP1 hyperactivation that degrades NAD^+^, causing loss of ATP^31^. Stable shOGG1 depletion significantly rescued the severe PARP1 hyperactivation-mediated ATP loss in ß-lap-exposed MiaPaca2 or ASPC1 PDA cells (Fig. 2D,E). Rucaparib (25 μM, 2 h pretreatment) addition, a PARP1 catalytic inhibitor, prevented short-term (within 2 h) ß-lap-induced programmed necrosis monitored by ATP loss (Fig. 2D,E), consistent with a role of PARP1 hyperactivation in ß-lap-induced programmed necrosis previously described by our lab^32^; exposure of cells to 25 μM Rucaparib for 2 h did not cause growth suppression, cell cycle changes or lethality.

XRCC1 acts as an integral scaffolding node to mediate BER signaling at the sites of DNA lesions, typically an AP sites or SSBs^21^. To elucidate the role of XRCC1 in ß-lap-induced lethality, we generated 10 stable shXRCC1 clones varying in expression (e.g., scrambled control and shXRCC1#1–8 are shown) (Fig. 2F). shXRCC1-depleted cells did not display altered baseline growth characteristics compared to parental or shRNA-Scrambled (shScr) control cells. When MiaPaca2 knockdown clones were ranked by relative XRCC1 expression (using actin for normalization), stable loss of relative XRCC1 protein expression strongly correlated with hypersensitivity to ß-lap, relative to non-targeted shScr control cells (Fig. 2G). Using stable shXRCC1 clone #8, whose levels were depleted >90% by Western blotting (Fig. 2F,G), we showed significant hypersensitivity to ß-lap in a dose-dependent manner (Fig. 2H). Loss of XRCC1 dramatically increased DSB formation as measured by γH2AX foci after ~15 min of treatment with ß-lap. Delayed DSBs were formed in a dose-dependent manner compared to similarly treated stable shScr MiaPaca2 cells (**SF3B**). Furthermore, stable XRCC1 depletion dramatically sensitized cells to ß-lap-induced NAD^+^ and ATP losses (Fig. 2I**, SF3B**), where degradation of intracellular NAD^+^ and indirectly ATP pools, were surrogates for PARP1 hyperactivation^8^,^13^. These data strongly suggested that loss of XRCC1 promoted prolonged AP sites and/or SSBs initially created by ß-lap that, in turn, cause PARP1 hyperactivation. These data are consistent with known kinetics and interactions of XRCC1 and PARP1 at DNA lesions, where XRCC1 aids disassociation of PARP1 from damaged sites and attenuates further PARylation allowing other BER factors access for DNA repair function (**SF2A**)^15^,^21^. Interestingly, ß-lap-treated XRCC1-depleted cells exhibited decreased metabolic recovery after PARP1 hyperactivation, as indicated by significant decreased ATP recovery over a 4 h period, and decreased lactate accumulation (an indicator of glycolytic activity) over a 6 h period after drug treatment (**SF3C, SF3D**). XRCC1 depleted cells did not show alterations in growth or cell cycle distribution compared to shScr containing MiaPaca2 cells. Overall, these data suggest that initial NAD^+^ depletion via hyperactivated PARP1 led to intracellular metabolic catastrophe from which cells were unable to recover.

While there are currently no known XRCC1 inhibitors, we wondered whether XRCC1 expression was decreased (a genetic vulnerability) in PDA tissue *vs* associated normal pancreas tissue in patient samples, as noted in head and neck cancers^33^,^34^,^35^. XRCC1 loss would provide a strategy to exploit a specific PDA vulnerability, and further increase the tumor selectivity of ß-lap in these cancers by the mechanism above. We compared XRCC1 mRNA expression levels in matched PDA *vs* associated normal pancreas tissue isolated from 45 PDA patients^36^ and discovered that XRCC1 expression was significantly elevated in PDA tissue relative to associated normal pancreatic tissue. These data suggest that at least a subset of PDAs may mount a robust BER resistance response to ß-lap exposures *vs* associated normal pancreas tissue (**SF3F**). While the significance of XRCC1 elevations in PDAs not known, we reasoned that pharmacological inhibition of BER in PDA, instead of segregating PDA patients based on XRCC1 expression, would be the most practical strategy to enhance tumor specificity and efficacy of ß-lap (ARQ761).

### The AP site modification factor, methoxyamine (MeOX), sensitizes cancer cells to ß-lap in an NQO1-dependent manner

**Figure 3:**
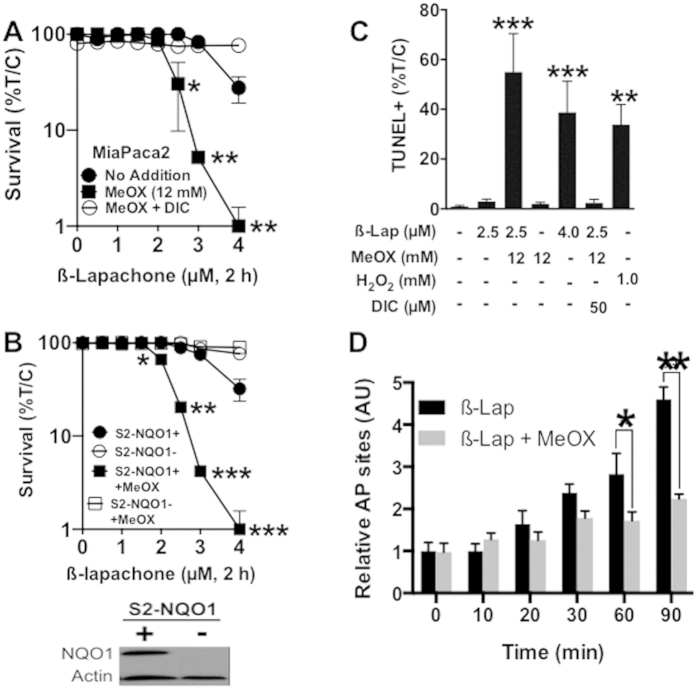
The AP site modifying factor, Methoxyamine (MeOX), sensitizes cancer cells to ß-lap in an NQO1-dependent manner. (**A**,**B**) Clonogenic survival of MiaPaca2 or NQO1+ S2-NQO1(+) *versus* NQO1-deficient S2-NQO1(−) cells treated with ß-lap, ±12 mM MeOX, with or without 50 μM DIC for 2 h. Data represent survival means ± SE from sextuplicate samples. (**C**) Quantification of TUNEL+ MiaPaca2 cells after ß-lap ±12 mM MeOX, with or without 50 μM DIC after 2 h. (**D**) AP sites were monitored in MiaPaca2 cells exposed as in ‘C’ using a reactive aldehyde probe over time. Note that MeOX-modification of AP sites hides AP site measurements using the reactive aldehyde probe.

MeOX covalently binds to aldehydes exposed within AP sites and the subsequent modified MeOX-AP site prevents access of BER complexes to the damage^37^,^38^,^39^,^40^,^41^. Increased levels, and prolonged half-lives, of AP sites result. Although topoisomerase IIα was implicated in the repair of modified AP sites^9^, it was not known whether these DNA lesions would promote or prevent PARP1 binding and hyperactivation. MiaPaca2 and genetically matched SUIT2-NQO1-deficient (S2-NQO1-) and SUIT2-NQO1-proficient (S2-NQO1+) metastatic PDA cells were treated with various ß-lap doses (μM, 2 h), ± 12 mM MeOX, and with or without Dic for 2 h (Figs. 3A,B). Combined MeOX + ß-lap treatment led to significant synergistic lethality *vs* ß-lap or MeOX alone, but only in NQO1-expressing cells (Fig. 3B). The lethality of ß-lap alone (not shown) or the combination of MeOX + ß-lap was rescued by inhibiting NQO1 using Dic (Fig. 3A,B). MeOX alone (12 mM, 2 h) did not affect cell viability, plating efficiency or survival (Fig. 3A). A sublethal dose of ß-lap (2.5 μM, 2 h) in combination with a nontoxic dose of MeOX (12 mM) led to dramatic increases in %TUNEL+ cells comparable to a lethal dose of ß-lap alone (4 μM, 2 h) or supralethal H_2_O_2_ exposures (1 mM, 30 mins). Synergy of MeOX + ß-lap was NQO1-dependent, since Dic addition completely blocked lethality of the combination as with ß-lap alone (Fig. 3C). To confirm that MeOX was hitting its target within the cell, we monitored AP site formation using a reactive aldehyde probe that competes with MeOX for AP site aldehyde binding. In this assay, modified MeOX-bound-AP sites are not detected by the probe. MeOX co-treatment blocked detection of AP sites by the probe generated from ß-lap treatment in NQO1+ MiaPaca2 cells (Fig. 3D), consistent with the published mechanism of action of MeOX^39^,^41^,^42^. In contrast, exposure to ß-lap alone generated over 5-fold increases in AP sites within a 90 min period (Fig. 3D).

We then examined survival following treatment with ß-lap ± 12 mM MeOX in a broad range of NQO1-expressing *vs* genetically matched NQO1-deficient cancer cell lines, previously validated for NQO1 status (Table 1)^6^,^26^,^29^. Complete dose-response analyses revealed a consistent LD_50_ dose enhancement ratio (DER) of >1.7 when NQO1+ breast, non-small cell lung, head and neck, and S2-NQO1+/− (Fig. 3B) PDA cell lines were exposed to an LD_50_ of ß-lap (ranging from 1.8–3.0 μM for 2 h, depending on cell line) in combination with MeOX (12 mM, 2 h); DER values were calculated as the LD_50_ ratio of cells exposed to ß-lap alone divided by cells treated with ß-lap + MeOX (Table 1); MeOX treatments were nontoxic. For each cell line, lethality caused by ß-lap alone, or in combination with MeOX, was prevented by Dic addition (Table 1). In contrast, genetically matched NQO1-deficient cells remain nonresponsive to either ß-lap alone or ß-lap + MeOx, and DER value calculations were not applicable (NA) (Table 1).

### Methoxyamine potentiates ß-lap-induced PARP1-hyperactivation and delayed DSB formation

**Figure 4:**
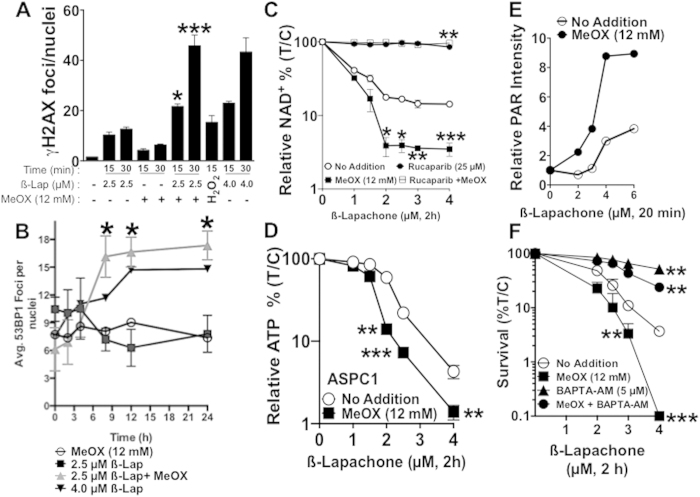
MeOX sensitizes cells to ß-lap by potentiating DNA damage and PARP1 hyperactivation. (**A**) MeOX enhances DSB formation in MiaPaca2 cancer cells. Average γH2AX foci/nuclei were assessed in ß-lap-treated (μM, 2 h) MiaPaca2 cells, + MeOX (12 mM) at 15 and 30 min during exposure. H_2_O_2_ (0.5 mM, 15 min) was used as a positive control. (**B**) Average 53BP1 foci/nuclei in ß-lap-treated (μM, 2 h) MiaPaca2 cells, ±12 mM MeOX over time (h). Graph represents average foci of three separate experiments. **(C)** Relative NAD^+^ levels in MiaPaca2 cells 2 h after treatment with ß-lap, ±12 mM MeOX, with or without Rucaparib (25 μM) in sextuplicate, means ± SEM. (**D**) Relative ATP levels in ASPC1 cells 2 h after treatment with ß-lap, ±12 mM MeOX. (**E**) Western blot quantification of relative PAR-PARP1 formation (using α-tubulin for loading) in MiaPaca2 cells 20 min after exposure to various doses of ß-lap ±12 mM MeOX. (**F**) Clonogenic survival of MiaPaca2 cells pre-treated with 5 μM BAPTA-AM (Ca^2+^ chelator) for 30 min followed by treatment with ß-lap (μM, 2 h), ±12 mM MeOX. Data represent survival means ±SEM from quadruplicate samples.

Using γH2AX as a proxy for DSB formation, we noted that treatment with a nontoxic MeOX dose (12 mM) + a sublethal ß-lap dose (2.5 μM, 2 h) significantly increased γH2AX formation in a delayed manner at 15 and 30 min *vs* ß-lap (2.5 μM, 2 h) or MeOX (12  mM, 2 h) alone (Fig. 4A). At 30 min, the combination was more efficient at inducing delayed DSBs than either a lethal dose of ß-lap (4 μM, 2 h) or a supralethal dose of H_2_O_2_ (1 mM, 15 min) alone. Using 53BP1 foci as an additional marker for DSB formation, we noted that DSB repair was severely impaired in cells exposed to ß-lap (2.5 μM) + MeOX (12 mM) for 2 h *vs* a sublethal dose of ß-lap (2.5 μM) alone (Fig. 4B, **24 h immunofluorescence image, SF4A**). A nontoxic dose of ß-lap alone caused damage after 2 h, but DNA lesions were quickly repaired, returning to basal levels in 9 h. In contrast, a sublethal ß-lap dose + a nontoxic dose of MeOX caused DSB formation equivalent to a lethal dose of ß-lap (4 μM, 2 h), and both treatments caused DSBs that were not repaired over a 24-h period (Fig. 4B). In contrast, although cells treated with a sublethal dose of ß-lap (2.5 μM) or a nontoxic MeOX dose (12 mM, 2 h) alone exhibited significant DSBs, these exposed cells were able to resolve their DSBs within 3 h post-treatment (Fig. 4B). Importantly, there was no significant difference in ROS formation in MiaPaca2 cells exposed to ß-lap alone *vs* ß-lap + MeOX exposures (**SF4B**), strongly suggesting that MeOX did not affect NQO1-dependent futile redox cycling, nor the level of initial DNA lesions formed.

We previously reported that ß-lap-induced lethality was driven by the dramatic loss of NAD^+^ pools as a consequence of PARP1 hyperactivation in a broad range of NQO1-expressing cancer cells^8^,^28^. To indirectly determine the effect of MeOX on ß-lap-induced threshold levels of DNA lesions required for PARP1 hyperactivation, we assessed changes in relative NAD^+^/ATP pools after various doses of ß-lap (μM, 2 h), in the presence or absence of an otherwise nontoxic dose of MeOX (12 mM, 2 h). MeOX addition dramatically decreased NAD^+^ pools in MiaPaca2 cells and ATP pools in ASPC1 cells in a ß-lap dose-dependent manner up to 2 μM ß-lap, and within 2 h co-treatment compared to ß-lap alone (Fig. 4C,D). Importantly, addition of a PARP1 inhibitor (e.g., Rucaparib) significantly blocked NAD^+^ depletion after either ß-lap alone or the combination therapy (Fig. 4C,D). Assessment of PAR polymer formation after 20 min of treatment with various doses of ß-lap ± MeOX revealed that PAR formation increased at significantly lower doses of ß-lap when MeOX was added compared to ß-lap exposure alone (Fig. 4E**, SF5A**). These data strongly suggest that addition of MeOX lowered the threshold level of DNA damage created by ß-lap required for PARP1 hyperactivation, presumably by inhibiting repair and prolonging MeOX-AP site formation. Since endoplasmic reticulum released calcium (Ca^2+^_ER_) is required for PARP1 hyperactivation after ß-lap treatment^7^,^8^,^27^, we noted significant sparing of ß-lap-induced lethality after BAPTA-AM addition, which chelates intracellular Ca^2+^. Colony formation assays showed that pre-loading cells with BAPTA-AM (5 μM, 2 h) offered significant protection of cells from ß-lap-induced lethality, with or without MeOX co-treatment (Fig. 4F).

### Methoxyamine potentiates PARP1-dependent metabolic catastrophe

**Figure 5:**
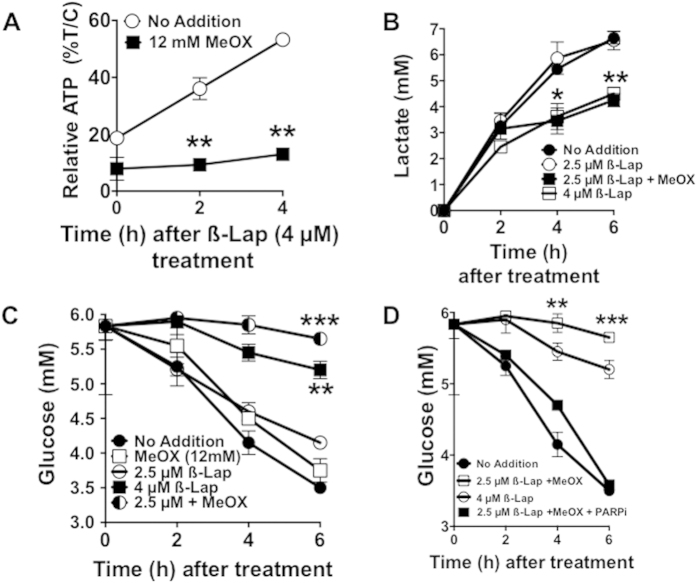
Methoxyamine potentiates ß-lap-induced PARP1-dependent metabolic catastrophe. (**A**) Relative ATP recovery in MiaPaca2 cells after a 2 h treatment with or without ß-lap, ±12 mM MeOX assessed over a 4 h time period by CellTiter-Glo assays. (**B**,**C**) Time-course of lactate production and glucose consumption over a 6 h period in media of MiaPaca2 cells after a 2 h treatment with ß-lap, ±12 mM MeOX. (**D**) Glucose consumption assessments from media of MiaPaca2 cells treated with Rucaparib (25 μM) for 2 h and then exposed to ß-lap ±12 mM MeOX for 2 h. Data represent means ±SEM from triplicate samples. All results were compared using Student’s t-tests (+/− standard deviation) unless otherwise stated. *p *<* 0.05; **p *<* 0.01; ***p *<* *0.001.*

ATP recovery after ß-lap treatment was blunted after co-treatment with MeOX compared to ß-lap alone (Fig. 5A). Additionally, when combined with a sublethal dose of ß-lap, MeOX suppressed lactate production and glucose utilization over a 6 h period (Fig. 5B,C). Consistent with a role of PARP1 hyperactivation in ß-lap-induced NAD^+^ loss (Fig. 4C), addition of the PARP1 inhibitor, Rucaparib, significantly attenuated suppression of glucose utilization in ß-lap-treated MiaPaca2 cells, regardless of MeOX addition (Fig. 5D)^43^,^44^,^45^,^46^. Indeed, Rucaparib addition restored glucose utilization to all ß-lap-treated cells (**SF6**). Note that the combination of a sublethal ß-lap dose (2.5 μM, 2 h) + a nontoxic dose of MeOX (12 mM, 2 h) was far superior to ß-lap alone in suppressing glucose utilization in MiaPaca2 cells (Fig. 5C). MeOX alone treatments had no effect on glucose utilization compared to untreated cells (Fig. 5C).

### MeOX + ß-lap provides synergistic antitumor activity against human pancreatic cancer xenografts *in vivo*

**Figure 6:**
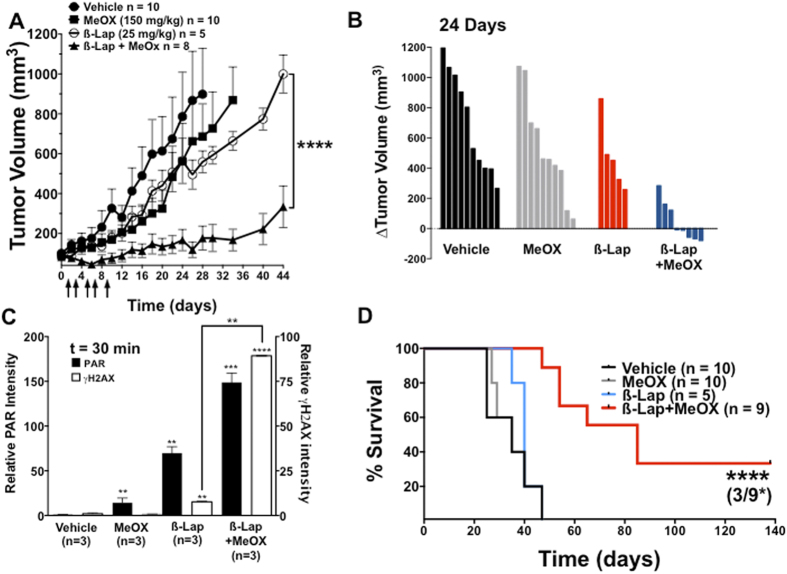
MeOX enhances ß-lap antitumor activity against subcutaneous human pancreatic xenografts. (**A**) Athymic nude mice bearing subcutaneous human MiaPaca2-derived xenograft tumors (100 mm^3^) were treated every other day with vehicle alone (i.p. HPßCD, n = 10), a nontoxic dose of MeOX (i.p., 150 mg/kg in saline, n = 10), a sub-efficacious dose of ß-lap (i.v., 25 mg/kg, n = 5) or nontoxic doses of MeOX + ß-lap (i.v., 25 mg/kg, n = 9) for a total of 5 doses over ten days (arrows). Tumor volumes (mm^3^) were monitored by direct caliper measurements. Tumor growth was monitored until tumor volumes reached 1,000 mm^3^, where necrotic tissue, restricted movement and weight loss warranted sacrifice. Error bars: means, +SEM. **(B)** Difference in individual tumor volumes from Day 0 and Day 24. Note regression of tumors in three mice from MeOX + ß-lap combination therapy. **(C)** Immunoblot quantification of relative PAR-PARP1 and γH2AX levels (with respect to α-tubulin loading) from tumors harvested 30 min after treatment with HPßCD vehicle alone, ß-lap alone, MeOX alone or ß-lap + MeOX at doses indicated in (**A**); n = tumors from 3 mice per group; Error bars: means, +SEM. (**D**) Kaplan-Meier survival plot of tumor–bearing mice treated with conditions described in (**A–C**). Mice were sacrificed when tumors reached 1,000 mm^3^ as per UT Southwestern Medical Center IACUC-approved animal protocol.

To determine if BER inhibition by MeOX would increase ß-lap-induced antitumor efficacy, including tumor regression *in vivo*, we generated subcutaneous xenografts in female Nu/Nu-athymic mice and treated these animals with one regimen as described^47^. This model was chosen since optimal ß-lap treatment regimen are typically minimally efficacious against subcutaneous xenografts at doses that are significantly lower than the MTD of 40 mg/kg HPßCD-ß-lap, i.v. alone^26^. Once MiaPaca2 xenografts were ~100 mm^3^, animals were treated every other day for 10 days with a total of five injections of: (**a**) sub-efficacious HPßCD-ß-lap (25 mg/kg, intravenous (i.v.)); (**b**) intra-peritoneal (i.p.) doses of MeOX (150 mg/kg); (**c**) a combination of ß-lap + MeOX at these doses and routes of administration; or (**d**) vehicle (HPßCD) alone as described^13^. Mice were sacrificed when tumor volumes reached 1,000 mm^3^. The combination treatment (MeOX + ß-lap) significantly delayed tumor growth relative to either treatment alone, and led to 3 regressions by day 24. Combination treatments caused no significant decrease in mouse weights over time (Fig. 6A,B **and SF7A**). Importantly, there was no significant increase in Methemoglobinemia after drug combination, which is the dose-limiting toxicity noted in ß-lap (ARQ761), pre-clinically or clinically^48^. During and well after treatment (up to day 12), we noted significant tumor regression in 5 of 8 mice following MeOX + ß-lap, where regression was not observed after any of the other treatments (Fig. 6A,B). Note that neither of the treatments (ß-lap nor MeOX alone) caused significant antitumor responses alone (Fig. 6A). For target validation, tumors were harvested 30 min after treatments above and analyzed for PAR-PARP1 (PAR) and γH2AX formation as pharmacodynamic (PD) markers of efficacy as described^13^,^29^,^31^ (Fig. 6C**, SF7B,C**). Consistent with our results *in vitro*, PAR formation increased ~150-fold after exposure to the combination of a sub-efficacious dose of HPßCD-ß-lap + MeOX compared to vehicle alone, and two-fold above HPßCD-ß-lap alone. Importantly, DSB formation (monitored by γH2AX levels) was dramatically elevated (>10-fold) in tumors from mice receiving MeOX + HPßCD-ß-lap (Fig. 6C**, SF7B,C**) compared to HPßCD-ß-lap alone.

Combined treatment of sub-efficacious doses of HPßCD-ß-lap + non-toxic MeOX significantly prolonged the survival of tumor-bearing mice (Fig. 6D). Long-term, mice were sacrificed when body weight dropped by one-third of their original values due to tumor-associated cachexia and when tumor volume exceeded 1000 mm^3^, or when tumors were ulcerated or impeded normal motion as per IACUC policies. Treatment with sub-efficacious doses of HPßCD-ß-lap (25 mg/kg) or MeOX (150 mg/kg) alone did not result in a significant difference in overall survival compared to vehicle (HPßCD) alone groups, with a median survival of ~47 days (Fig. 6D). In contrast, the combined HPßCD-ß-lap + MeOX treatment group had a median survival time of 75 days, which was 35 days longer than the median survival of vehicle (HPßCD) alone, or MeOX (150 mg/kg) alone or HPßCD-ß-lap (25 mg/kg) alone. Taken together, our observations indicate that use of a nontoxic dose of MeOX in combination with a sub-efficacious dose of ß-lap (HPßCD-ß-lap) in NQO1-expressing tumors results in increased PARP1 hyperactivation, significantly elevated DSB formation, significant antitumor activity and regression is some animals, with significantly extended survival compared to agents alone. Indeed, ~33% (3 of 9) of MiaPaca2-bearing xenograft mice exposed to HPßCD-ß-lap + MeOX exhibited apparent cures of their tumors (Fig. 6D).

## Discussion

There is a desperate need for new efficacious therapeutic strategies to treat recalcitrant PDAs. The standard-of-care for PDA consists of a series of DNA damaging agents (e.g., gemcitabine, Nab-paclitaxel) that lack tumor selectivity, have narrow therapeutic windows, and quickly become ineffective due to intrinsic or acquired drug resistance^2^. The NQO1/Catalase ratios demonstrated in pancreatic cancers (Fig. 1A**, SF1**) revealed a dramatic potential therapeutic window that can be exploited by NQO1 bioactivatable drugs, such as ARQ761 (ß-lapachone), currently in clinical trials (NCT01502800). The high NQO1/Catalase ratios in pancreatic cancers strongly suggest that NQO1-overexpressing PDA cancer cells will respond to NQO1 bioactivatable drugs by producing supralethal doses of H_2_O_2_. There is still very little known as to why NQO1 is elevated in pancreatic cancers. Numerous reports suggest that NQO1 elevation is a by-product of mutant K-RAS-driven Nrf2 overexpression in cancer cells, while others suggest that increased oxidative stress and inflammation drives NQO1 expression during tumorigenesis by activating NF-κB signaling through p53 inhibition^49^,^50^,^51^.

Concomitant low expression of Catalase should make PDA tumors selectively sensitive to NQO1 bioactivatable drugs, with a general lack of significant resistance^29^. Even if particular PDA tumors express significant Catalase levels, the NQO1-mediated futile redox cycle that generates H_2_O_2_ can easily saturate endogenous Catalase activities in these cancers^6^. Low NQO1 expression and elevated Catalase levels in most normal tissue, particularly in associated normal pancreas and liver, offer significant protection from NQO1 bioactivatable drugs, as noted in ß-lap-treated mice (Fig. 6). However, current NQO1 bioactivatable drugs cause significant NQO1-independent, dose-limiting Methemoglobinemia in red blood cells that somewhat restricts specific regimen, limiting efficacy^48^. Strategies, such as using specific DNA repair inhibitors to lower the efficacious doses of NQO1 bioactivatable drugs, would significantly reduce the risk of hemolysis and Methemoglobinemia noted with ß-lap (ARQ761). Data presented here offer ‘proof of principle’ in achieving superior efficacy of NQO1 bioactivatable drugs against recalcitrant NQO1-overexpressing PDAs, as well as most solid cancers.

Our BER-focused, limited synthetic lethal siRNA screen identified XRCC1 and OGG1 as the most significant modulators of ß-lap-induced PARP1 hyperactivation and lethality. We also identified APE1, and current research in the lab is focusing on this process for improving ß-lap efficacy using small molecule inhibitors. siXRCC1 silencing sensitized PDA cells to PARP1 hyperactivation and ß-lap-induced lethality. In the presence of DNA base damage, PARP1 recruits XRCC1 through PAR-mediated signaling that, in turn, binds and recruits other BER pathway members^21^. However, it is unclear whether XRCC1 recruitment can initiate PARP1 dissociation from the damage site, or if auto-PARylation alone is sufficient for this dissociation^14^,^52^. Upon XRCC1 knockdown, we observed enhanced PARP1 activity upon ß-lap treatment, suggesting that PARP1 residence and activity at damage sites may be attenuated by the recruitment of XRCC1, and its loss increases residence time and overall activity. In contrast, siOGG1 depletion dramatically spared cells from ß-lap-induced lethality and blunted PARP1 hyperactivation. This was unexpected given prior literature that demonstrated that OGG1 increases tolerance to menadione-induced ROS formation and PARP1 activity^53^. However, OGG1 can directly stimulate PARP1 activity, independent of its function as a DNA N-glycosylase^54^. Additionally, we believe that the NQO1-dependent futile cycling of ß-lap generates sustained supralethal H_2_O_2_ not observed with menadione (NQO1 expression detoxifies this drug), generating far more oxidative base damage and AP sites to facilitate PARP1 binding. Thus, OGG1-induced AP sites appear necessary to signal to PARP1 hyperactivation required for ß-lap-induced NAD^+^-Keresis.

DNA repair inhibitors have been under development for well over 40 years, and all developed drugs commonly lack tumor-selectivity, even when combined with focused ionizing radiation (IR) therapy. DNA damaging agents that specifically target selectively chosen cancers are desperately needed. Our data strongly suggest that NQO1 bioactivatable drugs enable tumor-selective use of DNA repair inhibitors, such as MeOX. We demonstrated that combination therapy with sublethal doses of ß-lap and a nontoxic dose of MeOX synergistically enhanced cytotoxicity in PDA, NSCLC, breast and head/neck cancers (Fig. 3, Table 1). The observed synergy was NQO1-dependent, since NQO1-deficient cell lines or dicoumarol (Dic)-co-treated cells were spared ß-lap-induce lethality. Mechanistically, the observed synergistic response with ß-lap + MeOX was driven by enhanced ß-lap-induced PARP1 hyperactivation due to prolongation of AP sites modified by MeOX (Fig. 4). These findings strongly suggested that modification of AP sites by MeOX increases the ability of PARP1 to exhaust NAD^+^ pools after ß-lap treatment. Once NAD^+^ levels are exhausted, PARP1 cannot function properly to repair SSBs induced by ß-lap, resulting in their rapid conversion to DSBs, as indicated by the dramatic increase in γH2AX and 53BP1 foci formation. While further biochemical studies will elucidate the chemistry of MeOX-bound-AP-sites, we believe this complex facilitates higher affinity binding between PARP1 and an AP site, increasing PARP1 residence time and the enzyme’s PARylation activity. Indeed, we conducted preliminary docking studies and found that MeOX bound AP sites in dsDNA provide two new close range hydrogen-bonding opportunities for Ser274 in PARP1, which can significantly stabilize the PARP1-AP-site interaction (**SF8**). Ongoing studies are proceeding in our lab to understand what other cofactors (e.g., released intracellular Ca^+2^_ER_) are needed mechanistically for PARP1 hyperactivation, since BAPTA-AM can significantly suppress PARP1 hyperactivation after ß-lap treatments, with or without MeOX (Fig. 4F).

The combination treatment developed here using ß-lap + MeOX addresses issues associated with both agents alone. Most importantly, it offers broad NQO1-dependent, tumor-selectivity to BER inhibitors (e.g., MeOX), while eliminating dose-limiting hemolysis and Methemoglobinemia caused by higher ß-lap doses, otherwise required for efficacy. The antitumor efficacy data presented (Fig. 6) offer ‘proof of principle’ that the combination enhances efficacy at well-tolerated doses of ß-lap and at completely nontoxic doses of MeOX. Furthermore, the ß-lap + MeOX doses used in our animal studies are relevant to those achievable in patients^55^,^56^. A major advantage of ß-lap + MeOX-induced NAD^+^-Keresis is the lack of resistance mechanisms available to cancers to overcome this combination therapy. Metabolically depleting cancer cells of NAD^+^/ATP results in the irreversible loss of glycolysis^8^. This treatment strategy will be pursued in further preclinical studies to optimize potential clinical utility, and to further elucidate the pathways and mechanisms of NAD^+^-Keresis.

## Materials and Methods

### AP site detection

AP sites were measured using a well described abasic site assay and an aldehyde reactive probe reagent (Dojindo Molecular Technologies, Inc., Gaithersburg, MD) as described^20^. Briefly, DNA (15 μg) samples extracted from cells with or without various drug treatments as indicated were incubated with 1 mmol/L aldehyde reactive probe at 37 °C for 10 min. After precipitation with 100% ethanol, DNA was washed and re-suspended in Tris-EDTA buffer [10 mmol/L Tris-HCl, 1 mmol/L EDTA (pH 7.2)]. DNA samples were denatured at 100 °C for 5 min, quickly chilled on ice, and mixed with an equal amount of ammonium acetate (2 mol/L). ssDNA was then immobilized on a BAS-85 NC membrane (Schleicher & Schuell, Dassel, Germany) using a vacuum filter device (Schleicher & Schuell). NC membranes were incubated with streptavidin-conjugated horseradish peroxidase (BioGenix, San Ramon, CA) at room temp for 30 min. After washing with buffer containing NaCl (0.26 M), EDTA (1 mmol/L), Tris-HCl (20 mmol/L), and Tween 20 (1%), aldehyde reactive probe-AP-sites were visualized with enhanced chemi-luminescence reagents (Amersham).

### Chemicals and Reagents

ß-Lap was synthesized and purified by us and stock solutions prepared at 50 mM in DMSO. Methoxyamine, dicoumarol, Rucaparib and BAPTA-AM were purchased from Sigma-Aldrich (St. Louis, MO).

### Cell Culture, siRNA silencing and drug treatments

All cell lines were obtained from the ATCC (Boulevard Manassas, VA) and grown in DMEM (Life Technologies, Carlsbad, CA) containing 10% FBS (Fisher Scientific, Waltham, MA) in a 5% CO_2_-95% air incubator at 37 °C. Cells were tested monthly and were free of mycoplasma contamination. Lipofectamine RNAiMAX (Life Technologies) was used for all siRNA transfections. Cells were transfected with one of two siRNAs purchased from Sigma-Aldrich to target OGG1 (OGG1 #1: SASI_Hs02_00340191, OGG1 #2: SASI_Hs02_00340192), or a non-targeting control siRNA). After 48 h of incubation with RNAiMax and siRNA in OptiMEM (Life Technologies), cells were detached with trypsin/EDTA (Life Technologies) and seeded for drug treatments or lysed for steady state protein silencing efficiency by Western blotting analyses. For drug combination treatments, cells were co-treated with ß-lap and 12 mM MeOX for 2 h in complete media as indicated. After treatment, drug-containing media were removed and replaced with fresh media.

### Colony forming ability assays

After ß-lap, MeOX, or MeOX + ß-lap treatments of cells at ~60% confluence, cells were trypsinized, counted by Coulter Counter, and diluted in single cell suspension. Cells were then seeded at 100, 500, or 1000 cells/plate on 60 mm dishes and allowed to grow for seven days. Plates were washed in PBS and colonies fixed and dyed with 3:1 methanol:crystal violet. Colonies of fifty or more normal-appearing cells were counted and results normalized to control colonies without drug treatment^5^.

### Glycolytic flux

A Seahorse XF24 bioanalyzer (Seahorse Bioscience, North Billerica, MA, USA) was used for mitochondrial stress tests. Cells were seeded at 3 × 10^4^ cells/well in 24-well plates washed with fresh Seahorse media. The stress test kit was used to inject oligomycin, FCCP, rotenone and 4 μM ß-lap at the indicated time points.

### Pooled RNAi synthetic lethal screen

MiaPaca2 cells were reverse transfected with 20 nmol/L of human On-Target Plus Smart pool using RNAiMAX for: XRCC1 (L-009394-00-005), OGG1 (L-005147-00-0005), FEN1 (L-010344-00-0005), APE1 (L-010237-00-0005) and MPG (L-005146-00-0005). After siRNA treatment for 48 h, cells were treated with ß-lap for 2 h, washed with PBS and plated at 500 cells/well onto 6-well dishes for 7-day colony forming assays.

### NAD^+^/ATP Assessments

CellTiter-Glo (Promega, Madison, WI) was used for cell viability (24 h after treatment) and ATP assays at indicated time points before or after drug treatments (2 h), unless otherwise indicated. NAD/NADH-Glo assays were also purchased from Promega (Madison, WI, USA). CellROX Green was obtained from Life Technologies (Carlsbad, CA, USA). All raw luminescent values for treatment conditions were normalized to the signal from untreated cells (T/C). Standard curves were generated to ensure linearity as described by the manufacturer.

### Immunoblot analyses

Cells were lysed in ice-cold RIPA with protease and phosphatase inhibitors (Santa Cruz, Dallas, TX). Whole-cell extracts were prepared by centrifugation (14,000 × g, 15 min) to remove insoluble components. Protein concentrations were determined by BCA assays (Thermo Scientific, Waltham, MA) and loading volumes were normalized. Proteins were then separated by 8% or 4–20% gradient SDS-PAGE (Bio-Rad, Hercules, CA) and transferred to PVDF membranes. Primary antibodies for protein detection included: phospho-H2AX (γH2AX, JBW301, Millipore, Billerica, MA), PARP1 (F-2, Santa Cruz), PAR (Trevigen, Gaithersburg, MD), Actin (C-2, Santa Cruz), XRCC1 (mouse monoclonal, Abcam, Cambridge, UK), small subunit μ-calpain (EPR3324, Abcam). Primary hybridizations were performed in Sigma casein blocking buffer at 4 °C overnight. Secondary HRP-conjugated antibodies were incubated for 1 h at room temperature, followed by detection with SuperSignal West Pico (Thermo Scientific). Bands were quantified by mean intensity using NIH ImageJ, and normalized to actin.

### Glucose consumption and lactate production

Cells were co-treated with ß-lap, with or without methoxyamine (MeOX) for 2 h in complete media. After co-treatment, media was replaced with low glucose, phenol free DMEM (Invitrogen) with 5% FBS and collected at indicated times for analyses with a BioProfile Automated Analyzer (Nova Biomedical, MA). All results are normalized to cell number.

### Flow Cytometry

For TUNEL analyses, cells were co-treated with ß-lap, MeOX, or ß-lap + MeOX for 2 h. Drug-containing media were removed and cells incubated in fresh complete media for 48 h. Cells were trypsinized, and both adherent and floating cells collected and washed in 1% BSA in PBS. After fixing cells in 70% ethanol, samples were washed and re-suspended in BSA/PBS buffer containing propidium iodine and saponin. Cells were analyzed on a FACSAria (BD Biosciences, San Jose, CA) and cell cycle distributions were modeled and calculated in FlowJo.

### Human xenograft antitumor and pharmacodynamic (PD) assays

Athymic female Nu/Nu nude mice (18–20 grams) were commercially obtained (Harlan). Human xenografts were generated by injecting 2 × 10^6^ MiaPaca2 cells subcutaneously in PBS/Matrigel into 6-week-old mice. Tumors were measured at indicated times with digital calipers (Fisher Scientific), and tumor volumes calculated (length × width^2^ × 0.5). Treatments were initiated when subcutaneous tumors reached an average size of >100 mm^3^. Mice were treated with MeOX (i.p.), ß-lap (HPßCD-ß-lap, i.v., retro-orbital) or both or with vehicle alone (HPßCD; 1:9, v/v; Sigma-Aldrich) as a control. Treatment regimens consisted of a total of 5 doses of drug, with one dose administered every other day over a 10-day period. Mice bearing subcutaneous tumors were treated with nontoxic doses of MeOX (150 mg/kg), with or without a sub-efficacious dose of HPßCD-ß-lap (25 mg/kg). Mice bearing subcutaneous tumors were sacrificed when tumors reached >1,000 mm^3^. Mice were weighed 3 times per week during and after the drug-treatments, with no toxicities. All animal studies were performed in accordance to approved protocols by UT Southwestern Medical Center Institutional Animal Care and Use Committee (IACUC).

### Microarray data processing and analyses

Gene expression data series were retrieved from the Gene Expression Omnibus (GEO) database subject to the following criteria: study included pancreatic cancer tumor or cell lines of pancreatic origin, more than 10 samples in the full study, processed using the GeneChip Human Genome U133 Plus 2.0 expression array. A total of 11 series met these criteria and were included in the cohort for analysis: GSE2109, GSE8332, GSE9599, GSE15471, GSE16515, GSE16648, GSE17891, GSE21654, GSE22337, GSE22780, GSE32676. The assembled cohort included 164 pancreatic tumor samples, 69 normal pancreas (n = 233) and 73 pancreas cell line specimens, for a total of 306 specimens. Within this assembled cohort were 59 matched-pair specimens from three independent studies, in which biopsies were taken from both tumor and adjacent normal lung for each patient studied. The specimen data files included in the cohort were downloaded as raw CEL files for post-processing as indicated, following the standard gene expression data preparation workflow^57^. We used the R package aroma.affymetrix, which uses persistent memory to allow analyses of very large datasets. Data were processed using the linear model from RMA, then fit robustly using probe level models as described^58^. Probe level models were fit to RMA-background corrected and quantile normalized data to obtain gene-level summaries. Gene-level summarization used the standard CDF provided by Affymetrix. The Welch’s t-test for unequal variance was used to compute p-values for the difference in the means. All analyses were performed in R. Statistical tests were performed using base R statistical functions, graphics were generated using the ggplot2 graphics package.

### Tissue Procurement and Pathology

The UT Southwestern Simmons Comprehensive Cancer Center Tissue Procurement Core IRB (CR00011064/STU 102010-051(8843), approved May 4, 2015), a standing IRB for procurement of de-identified patient samples, was used to secure pathologically diagnosed PDA tissue and associated normal tissue. IRB (CR00010749/STU 042011-005, April 15, 2015, PI: Dr. David Gerber for ‘A Phase I dose escalation study of ARQ761 (ß-lapachone) in adult patients with advanced solid tumors’ was also used.

### Statistics

Unless otherwise noted, graphs were plotted as means, with bars denoting standard error. Curve fitting and calculation of LD_50_ values, ANOVA, and two-tailed Student t-tests for statistical significance with Holm/Sidak multiple comparison correction were performed in GraphPad Prism 6.

## Additional Information

**How to cite this article**: Chakrabarti, G. *et al.* Tumor-selective use of DNA base excision repair inhibition in pancreatic cancer using the NQO1 bioactivatable drug, ß-lapachone. *Sci. Rep.* **5**, 17066; doi: 10.1038/srep17066 (2015).

## Figures and Tables

**Table 1:** MeOX enhances the lethality of ß-lap in an NQO1-dependent manner in a broad range of human cancer cells.

Cell line	Cancer Type	No Addition	MeOX (12 mM)	Dicoumarol (50 μM)	DER at LD_50_
231-NQO1(−)	Breast	>10	>10	>10	NA
231-NQO1(+)	Breast	1.8 ± 0.2	0.85 ± 0.09**	>10	2.12
UM-SCC-104-NQO1(−)	Head and Neck	>10	>10	>10	NA
SqCC/Y1-NQO1(+)	Head and Neck	2.0 ± 0.15	1.2 ± 0.07***	>10	1.67
H596-NQO1(−)	NSCLC	>10	>10	>10	NA
H596-NQO1(+)	NSCLC	3 ± 0.3	1.7 ± 0.2*	>10	1.76

Values represent ß-lap LD_50_ in μM concentration. Genetically matched NQO1-overexpressing (NQO1+) *versus* NQO1-deficient (NQO1−) cancer cells were screened for sensitivity to ß-lap alone, MeOX alone or the ß-lap + MeOX combination. Breast cancer: MDA-MB-231 NQO1 proficient = 231-NQO1(+); MDA-MB-231 NQO1 deficient = 231-NQO1(−). NSCLC: H596; Head and Neck cancer: UM-SCC104 and SqCC/Y1. DER = dose enhancement ratio; DER = LD_50_ +MeOX / LD_50_ No addition. All results were compared using Student’s t-tests (+/−SD). *p *<* 0.05; **p *<* 0.01; ***p *<* *0.001.*
